# Risk factors and clinical consequences of side branch occlusion in left anterior descending bifurcation percutaneous coronary intervention: a validation study of the V-RESOLVE score

**DOI:** 10.3389/fcvm.2025.1648244

**Published:** 2025-07-18

**Authors:** Xi Wu, Mingxing Wu, Haobo Huang, Zhe Liu, He Huang, Lei Wang

**Affiliations:** Department of Cardiology, Xiangtan Central Hospital (The Affiliated Hospital of Hunan University), Xiangtan, Hunan, China

**Keywords:** left anterior descending artery, percutaneous coronary intervention, side branch occlusion, V-RESOLVE score, coronary bifurcation lesions

## Abstract

**Background/purpose:**

Side branch occlusion (SBO) remains a prevalent and clinically significant complication during percutaneous coronary intervention (PCI) for bifurcation lesions, particularly those involving the left anterior descending (LAD) artery. This retrospective study aimed to assess the incidence, identify independent predictors, and evaluate the clinical consequences of SBO in the context of LAD bifurcation PCI.

**Methods:**

We conducted a retrospective analysis of 553 patients who underwent PCI targeting LAD bifurcation lesions between 2018 and 2023. Comprehensive data encompassing clinical characteristics, angiographic findings, and procedural details were collected. The primary outcome was the occurrence of SBO, defined as a reduction in side branch TIMI flow following stent implantation. Multivariate logistic regression was applied to determine independent risk factors.

**Results:**

SBO occurred in 41 cases (7.4%). Multivariate analysis identified true bifurcation lesions (OR 1.221, *P* < 0.001), an increased main vessel to side branch (MV/SB) diameter ratio (OR 1.431, *P* < 0.001), and higher Visual estimation-based Risk prEdiction of Side branch OccLusion in coronary bifurcation interVEntion (V-RESOLVE) scores (OR 3.736, *P* = 0.001) as significant independent predictors. Patients with SBO showed reduced procedural success rates (82.9% vs. 94.7%, *P* = 0.007), a higher incidence of periprocedural myocardial infarction (14.6% vs. 3.5%, *P* = 0.003), and increased rates of in-hospital major adverse cardiovascular events (MACE) (17.1% vs. 5.3%, *P* = 0.007).

**Conclusions:**

SBO is a clinically impactful yet partially preventable event in LAD bifurcation PCI. Key contributors include anatomical complexity, suboptimal protection strategies, and underutilization of intracoronary imaging. The V-RESOLVE score proved to be a robust predictor and may serve as a valuable tool for pre-procedural risk stratification, facilitating more tailored and effective intervention strategies.

## Introduction

Coronary bifurcation sites are particularly prone to atherosclerotic plaque accumulation due to disturbed hemodynamics and turbulent shear forces, which elevate oscillatory shear stress in these regions. Consequently, bifurcation lesions are commonly identified during coronary angiography(CAG) (1). The intricate morphology and distinctive anatomical structure of bifurcation coronary lesions (BCL) present considerable technical difficulties during percutaneous coronary intervention (PCI), resulting in higher procedural risk and complication rates. As such, BCLs are recognized as one of the most challenging subsets in interventional cardiology, accounting for approximately 15%–20% of all coronary interventions (2).

Side branch occlusion (SBO) is one of the most concerning complications encountered during bifurcation PCI (3). Following stent deployment in the main vessel (MV), the reported incidence of SBO ranges between 7.4% and 16.7% (4, 5). This event can result in perioperative myocardial infarction (MI) and stent thrombosis, significantly elevating the risk of major adverse cardiovascular events (MACE) and adversely affecting patient outcomes (6, 7). Multiple procedural and anatomical factors contribute to SBO during bifurcation PCI, including side branch (SB) diameter stenosis greater than 50%, extended lesion length within the SB, MV proximal stenosis over 50%, thrombus displacement at the SB ostium, vasospasm, dissection, an elevated MV/SB diameter ratio, and a wide carina angle (5, 8).

Among bifurcation lesions, those involving the left anterior descending artery (LAD) are the most prevalent, typically affecting diagonal and septal branches. Compared with lesions in the left circumflex (LCX) or right coronary artery (RCA), interventions in the LAD region are more likely to compromise SB flow (9). Prior studies have reported severe outcomes associated with SB occlusion in LAD lesions, including ventricular septal rupture and cardiac rupture (10). However, dedicated research focusing specifically on LAD bifurcation lesions remains limited. This single-center cohort study aims to investigate the incidence and predictors of SBO during PCI in patients with LAD bifurcation lesions.

## 2 Materials and methods

### 2.1 Study participants

**Figure 1:**
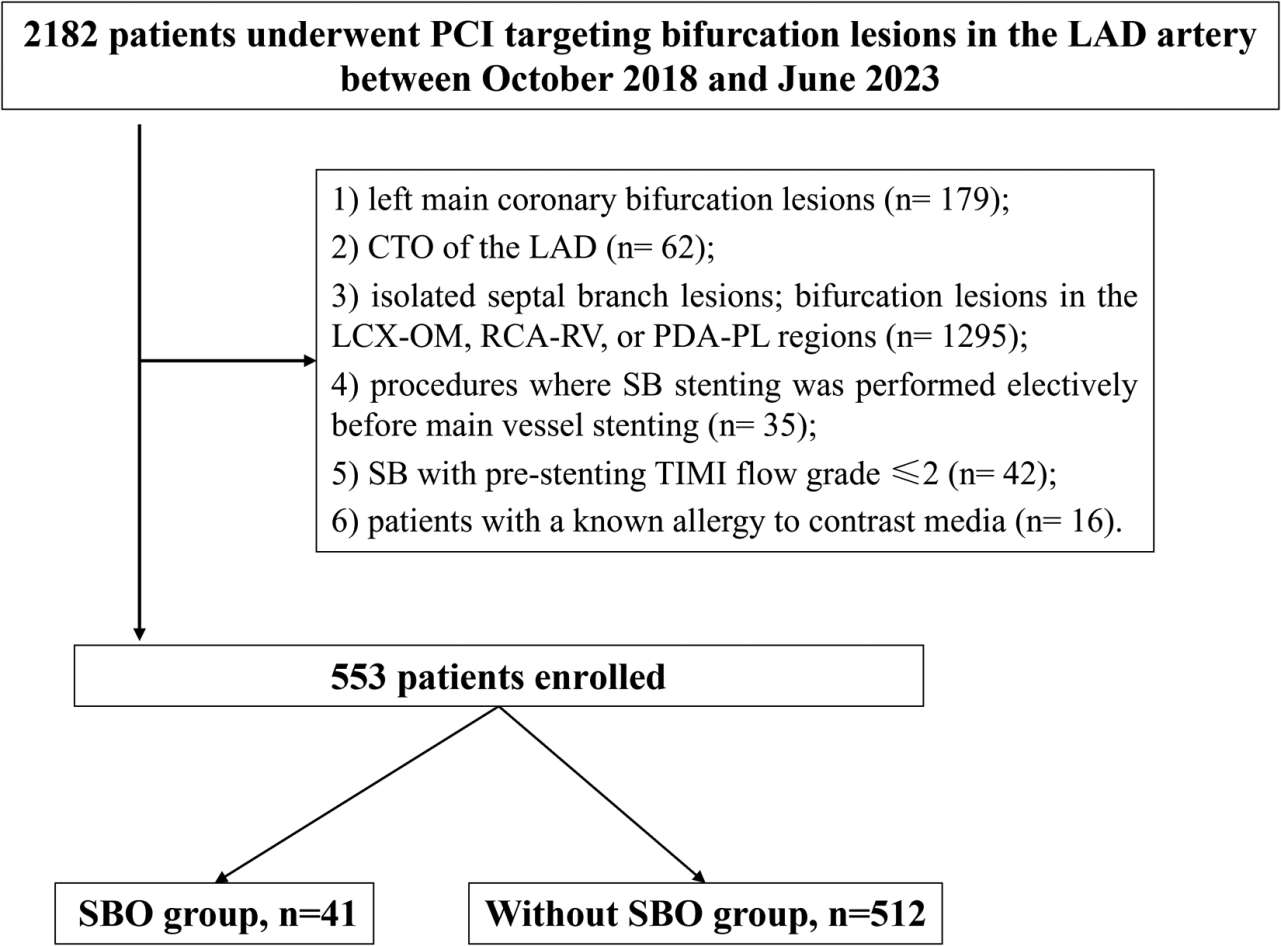
Study flow. PCI, percutaneous coronary intervention; LAD, left anterior descending artery; CTO, chronic total occlusion; LCX, left circumflex; RCA, right coronary artery; SBO, side branch occlusion.

This retrospective study enrolled patients who were admitted to the Department of Cardiology at Xiangtan Central Hospital between October 2018 and June 2023 for elective or urgent PCI targeting bifurcation lesions in the LAD artery, including presentations such as stable angina, unstable angina, non–ST-elevation myocardial infarction (NSTEMI), and ST-elevation myocardial infarction (STEMI). Prior to undergoing PCI, all participants provided written informed consent. The research protocol was approved by the Ethics Committee of Xiangtan Central Hospital and complied with the principles outlined in the Declaration of Helsinki (2013 revision). Ethical approval number: X201807352-1. Inclusion criteria were as follows: patients who underwent PCI for LAD bifurcation lesions with CAG confirming ≥70% diameter stenosis and a vessel diameter ≥2.5 mm; in addition, the implanted stent in the MV had to extend across the ostium of the SB. CBL were defined as atherosclerotic narrowing involving or located adjacent to the origin of a functionally significant SB. Exclusion criteria included: left main coronary bifurcation lesions; chronic total occlusion (CTO) of the LAD; isolated septal branch lesions; bifurcation lesions in the LCX-OM (obtuse marginal), RCA-RV (right ventricular), or PDA-PL (posterior descending artery- posterolateral branch) regions; procedures where SB stenting was performed electively before MV stenting; SB with pre-stenting TIMI (thrombolysis in myocardial infarction) flow grade ≤2; and patients with a known allergy to contrast media (Figure 1).

### 2.2 Data collection

Baseline demographic data, comorbid conditions, and laboratory parameters were systematically documented in a dedicated research database. The choice of vascular access for PCI (either radial or femoral), procedural strategy, device selection, and application of intracoronary imaging were left to the discretion of the interventional cardiologist, guided by current clinical practice guidelines and expert recommendations (11). All main vessel stents implanted were second-generation drug-eluting stents (DES), including everolimus-eluting and zotarolimus-eluting stents. Bare metal stents were not used in this cohort. Provisional stenting was the primary strategy utilized in this cohort, with SB stenting performed only if there was significant compromise following MV stenting. All participants received a loading dose of clopidogrel (300 mg) and aspirin (300 mg) within 24 h prior to the intervention. For P2Y12 inhibitor therapy, the majority of patients received clopidogrel (300 mg loading dose followed by 75 mg/day maintenance), while ticagrelor (180 mg loading dose followed by 90 mg twice daily maintenance) was administered to some acute coronary syndrome patients at the discretion of the treating physician. Prasugrel was not used in this cohort. During the procedure, unfractionated heparin was administered to maintain an activated clotting time within the therapeutic range of 250–300 s. Post-PCI, patients were prescribed dual antiplatelet therapy for at least 12 months, consisting of clopidogrel (75 mg once daily) or ticagrelor (90 mg twice daily) in combination with lifelong aspirin (100 mg/day).

### 2.3 Angiographic analysis

CAG was independently reviewed by two experienced investigators selected from a larger pool of qualified analysts. Offline assessment of the baseline angiographic images, obtained prior to PCI, was conducted using the QAngio software system (version 2.1.9, Medis, Leiden, the Netherlands). Quantitative coronary angiography (QCA) of both the MV and SB was performed according to standardized protocols previously reported in the literature (12).

The variables collected in this study encompassed four major categories: (1) Angiographic and procedural characteristics of the target bifurcation lesion, including the lesion's location within the main vessel lesion (ML), Medina classification, identification of true bifurcation lesions, stenosis severity at the bifurcation core, baseline bifurcation angle prior to PCI, and the total length of implanted stents. (2) ML–specific angiographic and procedural parameters, such as reference vessel diameter (RVD), degree of stenosis before the procedure, presence of moderate-to-severe calcification or angulation, evidence of thrombus, pre-PCI TIMI flow grade, plaque irregularity, dissection following pre-dilation, lesion preparation strategy (e.g., semi-compliant or non-compliant balloon use, with no systematic use of scoring balloons, cutting balloons, or intravascular lithotripsy recorded), post-dilation TIMI flow grade, and residual stenosis after pre-dilation. (3) SB–related angiographic and procedural characteristics, including reference diameter, pre-procedural stenosis severity, presence of moderate-to-severe calcification or angulation, thrombus involvement, TIMI flow grade before PCI, plaque irregularity, whether balloon pre-dilation was performed, use of the jailed wire technique, and residual stenosis after pre-dilation. (4) Primary outcome, defined as the occurrence of SBO following MV stent implantation.

### 2.4 Side branch occlusion management

In cases of SBO without a jailed wire in place, the side branch was first rewired, followed by balloon dilatation using semi-compliant or non-compliant balloons to restore flow. In cases with a jailed wire in place, the jailed wire was removed after the side branch was then rewired prior to balloon dilatation. If flow could not be adequately restored despite these measures, bailout side branch stenting was performed as a final strategy.

### 2.5 Definitions

Bifurcation classification: Among the various classification systems available for CBL, the Medina classification remains the most commonly adopted due to its simplicity, consistency, and ease of clinical application (13). A true bifurcation lesion was defined as one that met any of the following Medina criteria: 1,1,1; 1,0,1; or 0,1,1. MV plaque location: When atherosclerotic plaque was confined to the side of the MV opposite the SB ostium, it was classified as plaque opposite to the SB. If the plaque was situated on the same side as the SB ostium or simultaneously present on both sides, it was categorized as plaque on the SB side. Assessment of coronary artery calcification: The extent of calcification was evaluated based on standardized criteria previously established in the literature (14). Bifurcation angulation: The angle at the bifurcation site was categorized as follows: mild (<45°), moderate (>45° and <90°), or severe (>90°), based on the measured angle between the MV and SB. Coronary blood flow evaluation: Coronary perfusion was assessed using the TIMI flow grading system, in accordance with the definitions outlined by Gibson et al. (15). Coronary dissection was classified according to the criteria established by the National Heart, Lung, and Blood Institute (NHLBI) (16). SB pre-dilation referred to the use of balloon angioplasty in the SB prior to the implantation of the MV stent. Jailed wire technique was defined as the placement of a guidewire in the SB during MV stenting to safeguard patency at the SB ostium (2). The bifurcation core (carina region) was defined as the anatomical zone located within 5 mm proximal to the carina point, where the distal MV and SB intersect; this region was evaluated visually. Diameter stenosis (%): calculated as [RVD−minimal lumen diameter(MLD)]/RVD, representing the most severe luminal narrowing within the analyzed segment. MV/SB RVD ratio: calculated as (proximal MV reference diameter + distal MV reference diameter)/(2× SB reference diameter). Bifurcation angle (°) was defined as the angle formed between the central axis of the distal MV and that of the SB. RVD was visually estimated based on the dimensions of the proximal and distal artery segments not affected by atherosclerotic plaque, representing the presumed normal vessel caliber. SBO was defined as a complete or significant reduction in SB blood flow—either temporary or persistent—following MV stent implantation, characterized by a decline in TIMI flow grade (17). A SB was considered significant if its RVD, as assessed by QCA, was ≥1.5 mm. The Visual estimation-based Risk prEdiction of Side branch OccLusion in coronary bifurcation interVEntion (V-RESOLVE) score for each patient was calculated in accordance with the methodology and criteria established in prior studies (5). Derived from the Risk prEdiction of Side branch OccLusion in coronary bifurcation intervention (RESOLVE) study, the V-RESOLVE score is a simplified angiographic-based scoring system incorporating six bifurcation lesion characteristics to predict the risk of SOB during PCI. This scoring system was originally designed to estimate the likelihood of SBO in BCL. Technical success was defined by achieving TIMI grade 3 flow and a residual stenosis of less than 30% in both the MV and SB when SB stenting was performed. In cases where no SB stent was attempted, technical success was defined as TIMI grade 3 flow in the MV with residual stenosis <30%, and in the SB either (1) TIMI 3 flow with residual stenosis less than or equal to its baseline severity, (2) residual stenosis <50%, or (3) physiologically normal flow parameters. Procedural success was defined as the attainment of technical success without the occurrence of in-hospital MACE.

### 2.6 In-hospital major adverse cardiovascular events

In-hospital MACE included any of the following events occurring before discharge: cardiac death, MI, acute stent thrombosis, recurrence of symptoms requiring urgent repeat target-vessel revascularization (TVR) via PCI or coronary artery bypass grafting (CABG), and cardiac tamponade necessitating either pericardiocentesis or surgical intervention. All clinical endpoints and adverse outcomes were defined based on criteria provided by the Academic Research Consortium (18).

### 2.7 Statistical analysis

Statistical analyses were conducted using SPSS software version 26.0 (IBM Corp., Armonk, NY, USA). Categorical variables were summarized as frequencies and percentages, and intergroup comparisons were assessed using either the chi-square test or Fisher's exact test, depending on data distribution and sample size. Continuous variables were reported as mean ± standard deviation (SD) for normally distributed data, and as median with interquartile range (IQR) for data not following a normal distribution. For comparisons involving continuous variables, the independent samples *t*-test was applied when the assumption of normality was met; otherwise, the Mann–Whitney *U*-test was employed. To identify independent predictors of SBO, multivariate logistic regression analysis was performed. Variables included in the multivariate model were those demonstrating statistical significance in univariate analysis or deemed clinically important based on prior literature. A *P*-value less than 0.05 was considered to indicate statistical significance.

## 3 Results

### 3.1 Baseline clinical characteristics

A total of 2,182 patients were initially screened, of whom 553 met the inclusion criteria and were included in the final analysis (Figure 1). Among these, 41 cases (7.4%) experienced SBO. As presented in Table 1, baseline demographic characteristics and comorbid conditions were comparable between the SBO and without-SBO groups. The mean age did not differ significantly between the groups (57.3 ± 10.6 vs. 58.1 ± 11.6 years, *P* = 0.674), and the majority of patients in both cohorts were male (95.1% vs. 86.1%, *P* = 0.162). No statistically significant differences were observed in other clinical parameters, including body mass index, presence of diabetes mellitus, hypertension, dyslipidemia, smoking history, previous MI, stroke, peripheral artery disease, or left ventricular ejection fraction(LVEF) (all *P* > 0.05). Similarly, the distribution of clinical presentations—such as stable angina, unstable angina, STEMI, and NSTEMI—did not show a significant difference between the groups (*P* = 0.469).

**Table 1:** Baseline clinical characteristics.

Variables	All (*n* = 553)	SBO group (*n* = 41)	Without SBO group (*n* = 512)	*P*-value
Age, years	58.1 ± 11.5	57.3 ± 10.6	58.1 ± 11.6	0.674
Male sex, *n* (%)	480 (86.8)	39 (95.1)	441 (86.1)	0.162
BMI, kg/m^2^	24.6 ± 3.5	23.6 ± 2.9	24.6 ± 3.6	0.070
Diabetes mellitus, *n* (%)	127 (23.0)	13 (31.7)	114 (22.3)	0.234
Hypertension, *n* (%)	312 (56.4)	27 (65.9)	285 (55.7)	0.270
Dyslipidemia, *n* (%)	161 (29.1)	15 (36.6)	146 (28.5)	0.359
MI in 1 month, *n* (%)	100 (18.1)	8 (19.5)	92 (18.0)	0.971
Previous MI (>1 month), *n* (%)	125 (22.6)	7 (17.1)	118 (23.0)	0.492
Previous PCI, *n* (%)	152 (27.5)	9 (22.0)	143 (27.9)	0.520
Previous stroke, *n* (%)	70 (12.7)	4 (9.8)	66 (12.9)	0.736
Current smoking, *n* (%)	251 (45.4)	21 (51.2)	230 (44.9)	0.537
Previous peripheral vascular disease, *n* (%)	77 (13.9)	5 (12.2)	72 (14.1)	0.922
CAD presentation				0.469
Stable angina, *n* (%)	178 (32.2)	10 (24.4)	168 (32.8)	
Unstable angina, *n* (%)	216 (39.1)	15 (36.6)	201 (39.3)	
STEMI, *n* (%)	70 (12.7)	7 (17.1)	63 (12.3)	
NSTEMI, *n* (%)	89 (16.1)	9 (22.0)	80 (15.6)	
LVEF, %	59.8 ± 8.8	58.4 ± 5.7	60.0 ± 9.0	0.280

Continuous variables were expressed as mean ± SD. Categorical variables were expressed as number (percentage).

SBO, side branch occlusion; MI, myocardial infarction; PCI, percutaneous coronary intervention; NSTEMI, non-ST segment elevation myocardial infarction; STEMI, ST segment elevation myocardial infarction; LVEF, left ventricular ejection fraction.

### 3.2 Lesion and procedural characteristics

As shown in Table 2, the SBO group demonstrated significantly greater lesion complexity and procedural difficulty. True bifurcation lesions were markedly more prevalent among patients who developed SBO (68.3% vs. 35.5%, *P* < 0.001), with a higher incidence of Medina 1,1,1 configurations (53.7% vs. 25.4%, *P* = 0.004). Baseline angiographic assessments of both the MV and SB revealed notable disparities between the groups, particularly in terms of calcification severity, bifurcation geometry, and degree of ostial narrowing (Table 2). Within the MV, patients in the SBO group exhibited a higher frequency of moderate-to-severe calcification (41.5% vs. 29.5%, *P* = 0.034), thrombus-laden lesions (9.8% vs. 4.7%, *P* = 0.029), and irregular plaque morphology (14.6% vs. 6.1%, *P* = 0.028). Atherosclerotic plaque was significantly more likely to be situated on the same side as the SB ostium in these patients (90.2% vs. 35.2%, *P* < 0.001). Additionally, greater stenosis was observed in the bifurcation core (median 51.7% vs. 35.5%, *P* = 0.027), alongside narrower bifurcation angles (57.1° vs. 62.8°, *P* = 0.015), and elevated MV/SB reference diameter ratios (1.72 vs. 1.35, *P* = 0.041). In terms of SB characteristics, patients with SBO had smaller reference diameters (2.28 mm vs. 2.36 mm, *P* = 0.018) and more severe ostial stenosis (43.4% vs. 15.2%, *P* = 0.032). However, there were no significant group differences in SB calcification, angulation, thrombus presence, or pre-procedural TIMI flow grade (all *P* > 0.05). Procedural data further underscored the technical challenges associated with SBO. Dissection prior to MV stent implantation was more frequent in the SBO group (7.3% vs. 1.2%, *P* = 0.023). Notably, balloon pre-dilation of the SB was performed significantly less often in this group (4.9% vs. 25.4%, *P* = 0.005), and use of the jailed wire technique was considerably lower (14.6% vs. 43.0%, *P* < 0.001). Furthermore, patients who developed SBO had significantly higher V-RESOLVE scores (median 20.7 vs. 10.1, *P* < 0.001), and were substantially less likely to undergo pre-stenting intravascular imaging with intravascular ultrasound (IVUS) or optical coherence tomography (OCT) (2.4% vs. 33.2%, *P* < 0.001). Among the 41 cases defined as SBO, 39 patients (95.1%) successfully achieved TIMI grade 3 flow following side branch balloon dilatation. Bailout side branch stenting was required in 4 cases (9.8%) due to persistent flow compromise despite balloon dilatation, and 2 patients (4.9%) did not achieve full restoration of TIMI 3 flow even after stenting or balloon dilatation.

**Table 2:** Lesion and procedural characteristics.

Variables	All (*n* = 553)	SBO group (*n* = 41)	Without SBO group (*n* = 512)	*P*-value
Medina classification, *n* (%)				0.004
1,1,1	152 (27.5)	22 (53.7)	130 (25.4)	
1,1,0	175 (31.6)	7 (17.1)	168 (32.8)	
1,0,1	19 (3.4)	2 (4.9)	17 (3.3)	
0,1,1	39 (7.1)	4 (9.8)	35 (6.8)	
1,0,0	63 (11.4)	3 (7.3)	60 (11.7)	
0,1,0	91 (16.5)	2 (4.9)	89 (17.4)	
0,0,1	14 (2.5)	1 (2.4)	13 (2.5)	
True bifurcation lesion, *n* (%)	210 (40.0)	28 (68.3)	182 (35.5)	<0.001
Pre-procedural angiographic characteristics of MV				
RVD, mm	3.50 (3.36, 3.63)	3.50 (3.35, 3.67)	3.50 (3.36, 3.63)	0.628
Moderate to severe calcification, *n* (%)	168 (30.4)	17 (41.5)	151 (29.5)	0.034
Moderate to severe angulation, *n* (%)	145 (26.2)	13 (31.7)	132 (25.8)	0.336
Thrombus containing, *n* (%)	28 (5.1)	4 (9.8)	24 (4.7)	0.029
Pre-procedural TIMI flow grade, *n* (%)				0.984
TIMI 1 grade	24 (4.3)	2 (4.9)	22 (4.3)	
TIMI 2 grade	54 (9.8)	4 (9.8)	50 (9.8)	
TIMI 3 grade	475 (85.9)	35 (85.4)	440 (85.9)	
Irregular plaque, *n* (%)	37 (6.7)	6 (14.6)	31 (6.1)	0.028
Plaque location of MV, *n* (%)				<0.001
Opposite side of SB	336 (60.8)	4 (9.8)	332 (64.8)	
Same side of SB	217 (39.2)	37 (90.2)	180 (35.2)	
Stenosis of the diameter of the bifurcation core, (%)	36.30 (28.80–43.80)	51.70 (45.80–60.30)	35.50 (28.30–42.50)	0.027
Bifurcation angle,°	62.60 (53.90–71.10)	57.10 (45.00–66.80)	62.80 (54.30–71.80)	0.015
MV/SB reference vessel diameter ratio	1.37 (1.22–1.50)	1.72 (1.51–1.82)	1.35 (1.20–1.48)	0.041
Pre-procedural angiographic characteristics of SB				
RVD, mm	2.34 (2.17, 2.53)	2.28 (2.15, 2.35)	2.36 (2.17, 2.55)	0.018
Stenosis of the diameter of ostial SB, %	16.20 [7.60, 24.90]	43.40 [34.70, 52.60]	15.15 [7.10, 22.70]	0.032
Moderate to severe calcification, *n* (%)	1 (0.2)	0 (0.0)	1 (0.2)	0.619
Moderate to severe angulation, *n* (%)	26 (4.7)	2 (4.9)	24 (4.7)	0.545
Thrombus containing, *n* (%)	1 (0.2)	0 (0.0)	1 (0.2)	0.884
Pre-procedural TIMI flow grade, *n* (%)				0.236
TIMI 1 grade	0 (0.0)	0 (0.0)	0 (0.0)	
TIMI 2 grade	19 (3.4)	2 (4.9)	17 (3.3)	
TIMI 3 grade	534 (96.6)	39 (95.1)	495 (96.7)	
Irregular plaque, *n* (%)	10 (1.8)	1 (2.4)	9 (1.8)	0.764
Procedural characteristics				
MV				
Dissection before MV stenting, *n* (%)	9 (1.6)	3 (7.3)	6 (1.2)	0.023
TIMI flow grade after MV stenting, *n* (%)				0.268
TIMI 1 grade	0 (0.0)	0 (0.0)	0 (0.0)	
TIMI 2 grade	12 (2.2)	1 (2.4)	11 (2.1)	
TIMI 3 grade	541 (97.8)	40 (97.6)	501 (97.9)	
SB				
SB pre-dilation, *n* (%)	132 (23.9)	2 (4.9)	130 (25.4)	0.005
Jailed wire in SB, *n* (%)	226 (40.9)	6 (14.6)	220 (43.0)	<0.001
V-RESOLVE score	10.30 [9.20, 11.30]	20.70 [18.20, 21.70]	10.10 [9.10, 11.10]	<0.001
Pretreatment IVUS/OCT, *n* (%)	171 (30.9)	1 (2.4)	170 (33.2)	<0.001

Continuous variables were expressed as mean ± SD, or median (interquartile range). Categorical variables were expressed as number (percentage).

SBO, side branch occlusion; RVD, reference vessel diameter; TIMI, thrombolysis in myocardial infarction; MV, main vessel; SB, side branch; OCT, optical coherence tomography; IVUS, intravascular ultrasound.

### 3.3 In-hospital clinical outcomes

**Figure 2:**
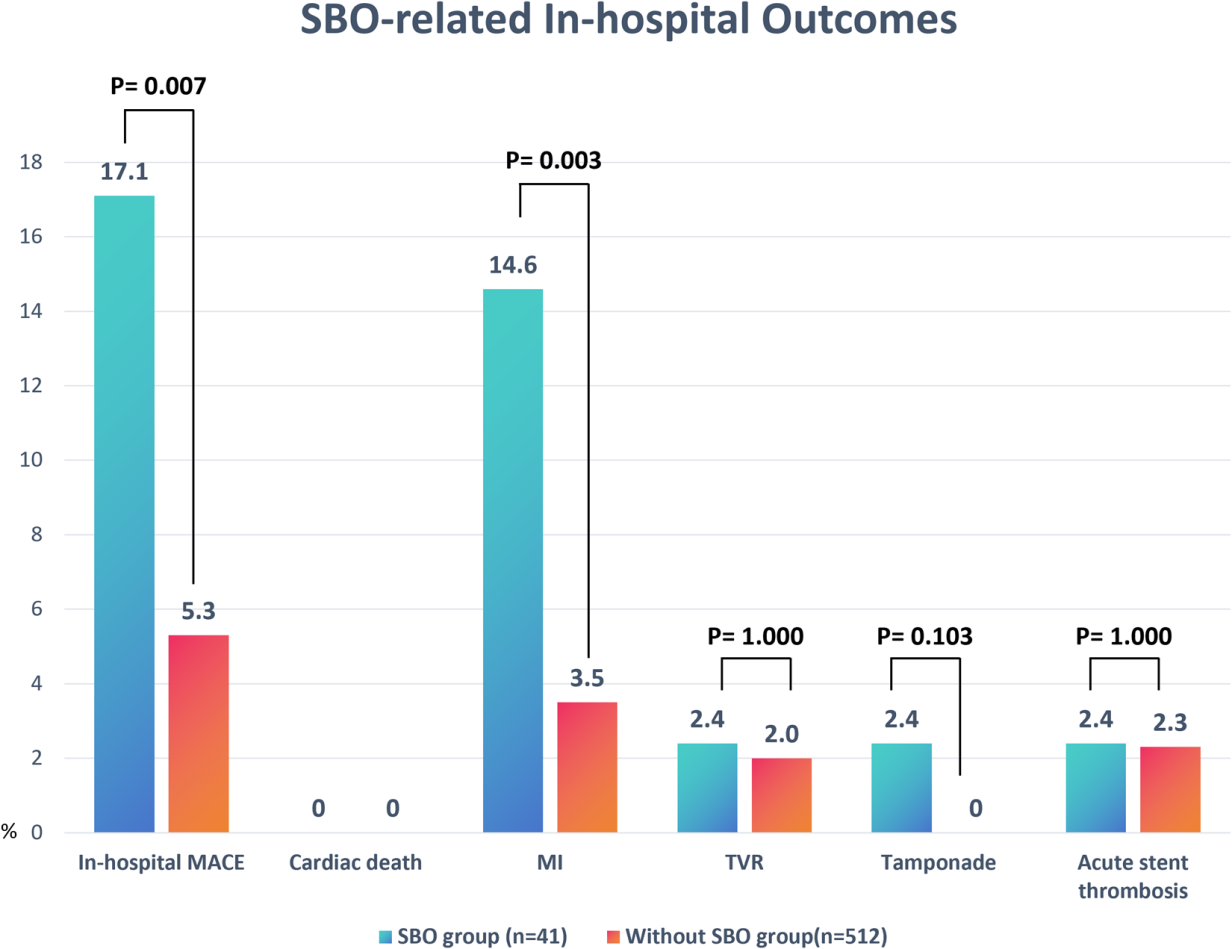
Comparison of in-hospital outcomes between patients with and without SBO. Bar chart illustrates the incidence rates (%) of MACE components, including cardiac death, MI, TVR, cardiac tamponade, and acute stent thrombosis, in the SBO group (*n* = 41) vs. the without-SBO group (*n* = 512). Significant differences were observed in the rates of overall in-hospital MACE (*P* = 0.007) and MI (*P* = 0.003). Data are presented as percentages. *P*-values were derived from chi-square or Fisher's exact tests as appropriate. SOB, side branch occlusion; MACE, major adverse cardiovascular events; MI, myocardial infarction; TVR, target vessel revascularization.

As detailed in Table 3 and Figure 2, the occurrence of SBO was significantly associated with a reduced rate of procedural success (82.9% vs. 94.7%, *P* = 0.007) and a higher incidence of in-hospital MACE (17.1% vs. 5.3%, *P* = 0.007). The increased MACE rate in the SBO group was primarily driven by a significantly elevated incidence of periprocedural MI (14.6% vs. 3.5%, *P* = 0.003). Other complications, including cardiac tamponade, acute stent thrombosis, and TVR, were observed infrequently and did not differ significantly between the two groups.

**Table 3:** Clinical outcomes and SBO-related in-hospital outcomes.

Variables	All (*n* = 553)	SBO group (*n* = 41)	Without SBO group (*n* = 512)	*P*-value
Procedural success, *n* (%)	519 (93.9%)	34 (82.9%)	485 (94.7%)	0.007
In-hospital MACE, *n* (%)	34 (6.1%)	7 (17.1%)	27 (5.3%)	0.007
Cardiac death, *n* (%)	0 (0)	0 (0)	0 (0)	-
MI, *n* (%)	24 (4.3%)	6 (14.6%)	18 (3.5%)	0.003
TVR, *n* (%)	11 (2.0%)	1 (2.4%)	10 (2.0%)	1.000
Tamponade, *n* (%)	1 (0.2%)	1 (2.4%)	0 (0.0%)	0.103
Acute stent thrombosis, *n* (%)	13 (2.4%)	1 (2.4%)	12 (2.3%)	1.000

Categorical variables were expressed as number (percentage).

SBO, side branch occlusion; MACE, major adverse cardiovascular event; TVR, target-vessel revascularization; MI, myocardial infarction.

### 3.4 Predictors of side branch occlusion

Univariate logistic regression analysis (Table 4) identified several factors significantly associated with the occurrence of SBO, including the presence of a true bifurcation lesion (*P* < 0.001), a higher MV/SB diameter ratio (*P* < 0.001), absence of jailed wire technique (*P* = 0.037), lack of SB pre-dilation (*P* = 0.022), and an elevated V-RESOLVE score (*P* = 0.001). Subsequent multivariate logistic regression analysis confirmed that three variables remained independent predictors of SBO: true bifurcation lesion [odds ratio [OR] 1.221, 95% confidence interval [CI] 1.052–1.522, *P* < 0.001], MV/SB reference diameter ratio (OR 1.431, 95% CI 1.333–2.727, *P* < 0.001), and V-RESOLVE score (OR 3.736, 95% CI 1.227–8.665, *P* = 0.001). These results emphasize the critical role of anatomical complexity and the importance of strategic procedural planning in reducing the risk of SBO during bifurcation PCI.

**Table 4:** Univariable and multivariable logistic analyses to predict side branch occlusion.

Variables	Univariate analysis			Multivariate analysis		
	OR	95% CI	*P*-value	OR	95% CI	*P*-value
Medina classification	1.302	0.640–3.179	0.455			
True bifurcation lesion	1.125	1.082–1.289	<0.001	1.221	1.052–1.522	<0.001
Moderate to severe calcification in pre-procedural of MV	1.233	0.737–3.824	0.327			
Thrombus containing in pre-procedural of MV	1.234	0.737–2.837	0.843			
Irregular plaque in pre-procedural of MV	1.622	0.737–2.827	0.528			
Plaque location of MV	1.624	0.729–4.521	0.178			
Stenosis of the diameter of the bifurcation core	1.410	0.736–1.730	0.631			
Bifurcation angle	1.672	0.836–3.626	0.073			
MV/SB reference vessel diameter ratio	1.626	1.466–2.771	<0.001	1.431	1.333–2.727	<0.001
RVD of SB	1.138	0.720–2.001	0.534			
Stenosis of the diameter of ostial SB	1.355	0.941–2.011	0.146			
Dissection before MV stenting	1.037	0.551–4.221	0.933			
SB pre-dilation	2.153	1.142–4.152	0.022	3.441	0.204–16.829	0.346
Jailed wire in SB	1.054	1.032–1.064	0.037	1.186	0.151–11.533	0.893
V-RESOLVE score	2.323	1.640–5.737	0.001	3.736	1.227–8.665	0.001
Pretreatment IVUS/OCT	1.023	0.445–2.335	0.602			

OR, odds ratio; CI, confidence interval; MV, main vessel; SB, side branch; OCT, optical coherence tomography; IVUS, intravascular ultrasound; RVD, reference vessel diameter.

## 4 Discussion

In this retrospective cohort study evaluating PCI for LAD artery bifurcation lesions, SBO was observed in 7.4% of cases. The results indicate that both anatomical and procedural variables play a crucial role in the development of SBO. Specifically, the presence of true bifurcation lesions, elevated MV/SB diameter ratios, and atherosclerotic plaques located on the same side as the SB ostium were significantly associated with increased SBO risk—highlighting the influence of lesion geometry on procedural outcomes. Additionally, insufficient SB protection strategies, including limited application of balloon pre-dilation and the jailed wire technique, as well as underuse of intravascular imaging modalities, emerged as modifiable technical contributors. Of particular note, the V-RESOLVE score demonstrated strong predictive capability for SBO, reinforcing its potential value as a pre-procedural risk assessment tool. SBO was also linked to a substantially higher incidence of in-hospital MACE, predominantly driven by periprocedural MI. These findings underscore the necessity for comprehensive anatomical evaluation and meticulous procedural planning to mitigate the risk of SBO and improve clinical outcomes.

### 4.1 Prevalence and anatomical determinants of side branch occlusion

In this single-center cohort study evaluating PCI for LAD artery bifurcation lesions, the incidence of SBO was 7.4%, consistent with previous studies such as the V-RESOLVE trial (7.4%) (5) and the COBIS II registry (8.4%) (8). These findings reinforce the reproducibility and clinical relevance of SBO as a common complication in bifurcation interventions. Although the PROGRESS-BIFURCATION registry reported a higher SBO incidence of 13%, the variation likely reflects differences in lesion complexity and inclusion criteria, with broader anatomical patterns represented in that cohort (19).

Several anatomical characteristics were found to be significantly associated with SBO in our study. True bifurcation lesions—especially those classified as Medina 1,1,1—were notably more common among patients who experienced SBO (68.3% vs. 35.5%, *P* < 0.001). This observation is consistent with prior research suggesting that true bifurcations are more vulnerable to plaque or carina shift during stent implantation in the MV (20). Additionally, atherosclerotic plaques positioned on the same side as the SB ostium were significantly more prevalent in the SBO group (90.2% vs. 35.2%, *P* < 0.001), a finding supported by OCT study showing that layered or bulky plaques at the SB origin increase the likelihood of occlusion (21).

Geometrical considerations also played a key role. A narrower bifurcation angle was independently associated with SBO in our population (57.1° vs. 62.8°, *P* = 0.015), likely due to increased susceptibility to carina displacement during stent expansion. Although the impact of bifurcation angle on SB outcome remains a topic of debate, several investigations support the notion that narrower angles elevate the risk of carina shift and subsequent SB occlusion (22). Conversely, some studies have suggested that wider bifurcation angles may also compromise SB outcomes by reducing ostial area or shortening ostial length (23). One analysis even found that the SBO group exhibited larger bifurcation angles than non-SBO counterparts (23). Nonetheless, our results align more closely with studies that highlight mechanical carina shift—more pronounced at narrower angles—as a key mechanism of SBO development (21).

Another important predictor was the MV/SB reference diameter ratio, which was significantly higher in the SBO group (1.72 vs. 1.35, *P* = 0.041). This metric reflects the relative vessel size mismatch and has been implicated in plaque redistribution and hemodynamic compromise at the SB ostium during stent deployment, as documented in IVUS-based investigations (24). Furthermore, a higher degree of stenosis within the bifurcation core was observed in SBO cases (51.7% vs. 35.5%, *P* = 0.027), emphasizing the contribution of lesion burden at the carina to impaired SB perfusion (20). Taken together, these anatomical risk factors highlight the critical importance of thorough preprocedural angiographic assessment in bifurcation PCI. They also provide a mechanistic framework suggesting that SBO is not merely a procedural mishap, but rather a foreseeable event stemming from adverse bifurcation geometry and complex plaque distribution.

### 4.2 Procedural factors and operator-dependent risk contributors

Beyond anatomical complexity, procedural techniques and operator-driven decisions play a pivotal role in determining the risk of SBO during bifurcation PCI. Our findings highlight several procedural characteristics that were significantly associated with SBO and may be amenable to optimization through refined interventional strategies.

Notably, SB pre-dilation was performed far less frequently in the SBO group compared to patients without SBO (4.9% vs. 25.4%, *P* = 0.005). Pre-dilation may enhance ostial lesion compliance and facilitate favorable plaque redistribution, thereby improving SB preservation during MV stenting. This protective role has been emphasized in prior studies, particularly for lesions with high plaque burden or tight ostial narrowing (20, 24). Similarly, use of the jailed wire technique—an established and technically straightforward approach to maintain SB access—was significantly lower in the SBO cohort (14.6% vs. 43.0%, *P* < 0.001). The presence of a jailed wire enables re-access and bailout maneuvers in cases of SB flow compromise and has been shown to lower the incidence of complete SB occlusion in high-risk bifurcation anatomy (24, 25).

Procedural complications also appeared to influence SBO risk. Dissections occurring prior to MV stent deployment were significantly more common among SBO cases (7.3% vs. 1.2%, *P* = 0.023), potentially contributing to altered flow dynamics or formation of a false lumen that impairs SB perfusion. These observations reinforce the importance of meticulous lesion preparation and conservative balloon sizing during pre-dilation and modification procedures (20, 24). A particularly striking finding was the markedly low rate of intravascular imaging use in the SBO group. Only 2.4% of these patients underwent IVUS or OCT guidance, compared to 33.2% in the without-SBO group (*P* < 0.001). Intravascular imaging provides valuable insights into lesion morphology, plaque eccentricity, and carina shift potential—factors that are critical in guiding stent deployment and minimizing SB compromise. Several studies have advocated for routine use of IVUS/OCT in complex bifurcation interventions to enhance procedural precision and outcomes (20, 24, 26). Taken together, these data suggest that SBO is not solely determined by anatomical constraints but is also significantly influenced by modifiable, operator-dependent factors. Inadequate SB preparation, failure to employ protective techniques, and underutilization of intravascular imaging are all preventable contributors. These findings support the need for greater procedural standardization and broader integration of evidence-based bifurcation techniques to reduce the incidence of SBO in contemporary practice.

### 4.3 Risk stratification with the V-RESOLVE score

Our study demonstrated a strong and statistically significant association between the V-RESOLVE score and the occurrence of SBO in patients undergoing PCI for LAD bifurcation lesions. Patients who experienced SBO had substantially higher V-RESOLVE scores compared to those without SBO (median 20.7 vs. 10.1, *P* < 0.001). Furthermore, multivariate logistic regression confirmed the V-RESOLVE score as an independent predictor of SBO (OR 3.736, 95% CI: 1.227–8.665, *P* = 0.001). These results support the V-RESOLVE scoring system's discriminatory power and validate its clinical applicability for peri-procedural risk stratification in real-world settings. This finding is consistent with the original V-RESOLVE study, which demonstrated that visual estimation of lesion characteristics could effectively substitute for QCA in predicting SBO, with a c-statistic of 0.76 (95% CI: 0.71–0.80)—a performance nearly equivalent to that of the QCA-based RESOLVE score (c-statistic: 0.77, 95% CI: 0.72–0.81) (5). Simulation analyses from the original study further showed acceptable inter-observer consistency (c-statistic range: 0.65–0.77), highlighting the score's robustness across different operators and institutions (5). Importantly, our analysis extends the validation of V-RESOLVE specifically to LAD bifurcation lesions—a subset characterized by higher SBO risk due to frequent involvement of critical diagonal or septal branches. While earlier studies have evaluated bifurcation PCI in general, few have focused exclusively on LAD-specific anatomy or validated risk assessment tools in this context. Our findings thus address a notable gap in the current evidence base. Given its predictive strength and practical simplicity, we advocate incorporating the V-RESOLVE score into routine pre-procedural assessments for bifurcation PCI. The score offers a rapid, visually assessed, and non-QCA-dependent tool to identify high-risk patients. This early stratification could inform the use of protective strategies—such as jailed wire or balloon techniques—or prompt the deployment of intravascular imaging to better characterize lesion morphology. When employed proactively, these measures have the potential to mitigate SBO risk and improve procedural outcomes (20).

Incorporating the V-RESOLVE score into standardized clinical workflows—potentially as part of a consensus-driven PCI decision-making pathway—could enhance both individualized treatment planning and institutional protocol consistency. While prior expert consensus documents have advocated for structured bifurcation PCI strategies, few have formally integrated validated predictive scores into these frameworks (20). Nevertheless, despite its utility, the V-RESOLVE score is subject to variability due to its reliance on visual assessment. To ensure consistency, operator training and standardized interpretation protocols are essential. Prospective, multicenter trials are warranted to further validate the prognostic impact of the V-RESOLVE score on long-term outcomes and to explore its integration within algorithmic, guideline-based approaches to bifurcation PCI.

### 4.4 Prognostic impact of SBO and clinical outcomes

SBO during PCI for bifurcation lesions carries significant clinical consequences and should no longer be viewed as a minor angiographic inconvenience. In our cohort of 553 patients undergoing PCI for LAD bifurcation lesions, SBO occurred in 7.4% of cases and was associated with notably lower procedural success (82.9% vs. 94.7%, *P* = 0.007), higher rates of periprocedural MI (14.6% vs. 3.5%, *P* = 0.003), and increased in-hospital MACE (17.1% vs. 5.3%, *P* = 0.007). These results highlight the immediate prognostic significance of SBO, even when the affected SB appears angiographically small or non-dominant.

Pathophysiologically, SBO impairs myocardial perfusion in the territory supplied by the occluded SB, potentially leading to ischemia, infarction, and microvascular dysfunction—particularly in cases involving functionally important branches such as diagonals or septals. Furthermore, the abrupt cessation of flow can result in endothelial damage and activate inflammatory cascades, exacerbating myocardial injury (11). Our findings align with previous research by Strepkos et al., who examined 933 bifurcation PCI cases across six centers. Their analysis demonstrated that SBO was linked to significantly lower procedural success (73.5% vs. 92.2%, *P* < 0.001), a greater need for unplanned two-stent strategies (24.8% vs. 6.0%, *P* < 0.001), and higher rates of dissection and plaque modification interventions (19). Importantly, patients with untreated SBO exhibited increased long-term MACE and mortality, emphasizing the enduring consequences of unaddressed SB compromise (19). Similarly, Guo et al. reported that in a cohort of 245 patients with chronic total occlusion and bifurcation lesions (CTO-BFL), SBO- defined as post-recanalization TIMI flow <3—was significantly associated with periprocedural MI and composite procedural complications. Key independent predictors included the absence of SB protection, ostial stenosis of the SB, and use of dissection-reentry techniques (27). Although some literature has suggested that occlusion of small or non-dominant SBs may be clinically inconsequential, our results and those from large registries challenge this assumption. Even SBs with reference diameters ≥1.5 mm can supply critical myocardial territories—particularly in LAD bifurcations—and their occlusion may initiate a cascade of ischemic injury (27). These findings reinforce the view that SBO is a clinically meaningful complication with both procedural and prognostic implications. Furthermore, periprocedural MI itself has been shown to be independently associated with increased short- and long-term all-cause mortality in patients undergoing PCI, underscoring its prognostic importance (28). It not only reflects underlying anatomical complexity but also acts as a direct mediator of myocardial injury and adverse outcomes. As such, prevention, early detection, and effective management of SBO should be prioritized in bifurcation PCI. Further prospective, large-scale studies are needed to investigate the long-term effects of SBO, including its role in target vessel failure, progression of heart failure, and mortality. Incorporating SBO risk assessment into pre-procedural planning and post-PCI surveillance protocols may contribute to improved short- and long-term cardiovascular outcomes.

### 4.5 Limitations

Several limitations of this study should be acknowledged. First, this was a single-center, retrospective cohort study, which may introduce inherent selection bias and limit the generalizability of our findings. Although multivariate analyses were performed to adjust for confounding variables, residual confounders cannot be fully excluded. Second, the assessment of lesion characteristics and V-RESOLVE scoring was based on angiographic visual estimation, which, despite standardization and operator training, is subject to inter-observer variability. Third, our study focused exclusively on LAD bifurcation lesions and may not be fully extrapolatable to left main or other coronary bifurcations. Fourth, the lack of long-term follow-up data precludes definitive conclusions regarding the chronic impact of SBO on clinical outcomes such as mortality, target vessel failure, or late stent thrombosis. Finally, although intravascular imaging was underused in the SBO group, its limited overall usage prevented subgroup analysis regarding its potential protective role. Future multicenter, prospective studies with comprehensive imaging and long-term outcome data are warranted to validate and extend these findings.

## 5 Conclusion

In this retrospective analysis of LAD bifurcation PCI, SOB occurred in 7.4% of patients and was significantly associated with adverse procedural and in-hospital outcomes, including increased rates of periprocedural MI and MACE. True bifurcation anatomy, high MV/SB diameter ratios, carina-adjacent plaque distribution, and lack of procedural protection emerged as key contributors to SBO. The V-RESOLVE score proved to be a powerful and independent predictor of SBO, supporting its integration into pre-procedural risk stratification workflows. Collectively, these findings highlight the need for meticulous anatomical assessment, risk-based planning, and adoption of evidence-based procedural strategies to minimize SBO risk. Future prospective studies are needed to validate imaging-guided and AI-assisted approaches for personalized bifurcation intervention planning.

## Data Availability

The raw data supporting the conclusions of this article will be made available by the authors, without undue reservation.
